# Motor Interaction Control Based on Muscle Force Model and Depth Reinforcement Strategy

**DOI:** 10.3390/biomimetics9030150

**Published:** 2024-03-01

**Authors:** Hongyan Liu, Hanwen Zhang, Junghee Lee, Peilong Xu, Incheol Shin, Jongchul Park

**Affiliations:** 1 Department of Marine Convergence Design Engineering, Pukyong National University, 45, Yongso-ro, Nam-Gu, Busan 48513, Republic of Korea; liuhongyan@pukyong.ac.kr (H.L.); zhanghanwen@pukyong.ac.kr (H.Z.); ljh@pknu.ac.kr (J.L.); 2 Department of Artificial Intelligence Convergence, Pukyong National University, 45, Yongso-ro, Nam-Gu, Busan 48513, Republic of Korea; 202356105@pukyong.ac.kr (P.X.); icshin@pknu.ac.kr (I.S.)

**Keywords:** muscle force modeling, deep reinforcement, motor interaction, stage particle swarm, environmental adaption

## Abstract

The current motion interaction model has the problems of insufficient motion fidelity and lack of self-adaptation to complex environments. To address this problem, this study proposed to construct a human motion control model based on the muscle force model and stage particle swarm, and based on this, this study utilized the deep deterministic gradient strategy algorithm to construct a motion interaction control model based on the muscle force model and the deep reinforcement strategy. Empirical analysis of the human motion control model proposed in this study revealed that the joint trajectory correlation and muscle activity correlation of the model were higher than those of other comparative models, and its joint trajectory correlation was up to 0.90, and its muscle activity correlation was up to 0.84. In addition, this study validated the effectiveness of the motion interaction control model using the depth reinforcement strategy and found that in the mixed-obstacle environment, the model’s desired results were obtained by training 1.1 × 10^3^ times, and the walking distance was 423 m, which was better than other models. In summary, the proposed motor interaction control model using the muscle force model and deep reinforcement strategy has higher motion fidelity and can realize autonomous decision making and adaptive control in the face of complex environments. It can provide a theoretical reference for improving the effect of motion control and realizing intelligent motion interaction.

## 1. Introduction

With the continuous development of robotics and its increasingly wide range of applications, motion interaction control shows great potential in the fields of healthcare, assisted living, and entertainment. However, a high degree of motor interaction control still faces many challenges [1]. As an important tool for studying human movement, muscle force modeling can simulate the characteristics of human muscles such as strength, range of motion, and force distribution [2,3]. Research on motion interaction control based on muscle force modeling can more accurately control the motion of robots and achieve a high degree of interaction with humans [4]. However, there are some shortcomings in the current motion interaction control model. The traditional motion control model is often only applicable to specific types of motion, unable to cover a variety of different forms of motion, which limits its application scope [5,6,7]. Therefore, improving the traditional motion control model to improve its performance and universality has become an urgent demand today. The deep reinforcement strategy is a method based on deep learning and reinforcement learning that has emerged in recent years with strong adaptability and generalization ability [8]. Research on motor interaction control based on deep reinforcement strategies can train a robot to interact with the environment so that it can learn the optimal behavioral strategies, thus achieving more accurate and efficient motor interaction [9]. Therefore, this study proposed a motor interaction control model based on a muscle force model and deep reinforcement strategy and used a staged particle swarm optimization (SPSO) algorithm to optimize the parameters in the model to improve the training efficiency [10]. This study aimed to realize autonomous decision making and adaptive interaction control of complex environments in the face of motion interaction models and provide a theoretical basis for further advancing the development of the field of motion interaction control. This study describes the current development of deep reinforcement learning algorithms and motor interaction control in Section 1 and constructs a motor interaction control model based on the muscle force model and deep reinforcement strategy in Section 2. In Section 3, the proposed reinforcement interaction motor control model is empirically analyzed. The conclusion and outlook of future research directions are presented in Section 4.

## 2. Literature Review

With the progress of social technology, deep reinforcement learning algorithms have been widely used in various fields. In order to optimize the energy utilization efficiency of electric buses and extend the power system life, Huang et al. proposed to construct an energy management model, validated the effectiveness of the model, and found that the model effectively extended the life of the battery and improved the efficiency of energy utilization, which, in turn, reduced the total operating cost, and the model made a contribution and had practical application value [11,12,13]. To optimize the computational performance and resource allocation capacity of a fog computing wireless access network, Jo et al. proposed to construct a computational task offloading and resource allocation strategy, validated the effectiveness of the proposed optimization strategy, and found that this strategy significantly improved the system processing efficiency and resource allocation compared with the traditional processing methods [14]. In the transportation system, the logistics path-planning performance is insufficient and there is the problem of long computation times, so Yu et al. proposed the deep reinforcement learning mechanism to optimize the logistics path-planning model to verify the model’s effectiveness, and found that the computation time of the model compared with the traditional model was significantly shortened, and the phase of the computation time in the path planning was better [15]. To further improve the precision and accuracy of fluid flow control, Rabault et al. proposed to apply deep reinforcement learning techniques to the training of the flow control to construct an active control model of the flow and validated the effectiveness of this model, which was found to successfully stabilize the vortex channel and reduce the resistance by approximately 8%, opening the way for the execution of active flow control [16,17,18]. In order to improve the elasticity of a power system, Sreedhar and others used a deep reinforcement learning algorithm to build a power system data-driven agent framework, whereby the validity of the method can achieve accurate system model calculations, overcome scalability problems, and enhance the deployment of power system elastic shunt planning [19]. In order to solve the problem of improving the system throughput and reducing the transmission delay of the multi-beam satellite system, Hu et al. proposed a black hole lighting plan optimization method based on deep reinforcement learning to verify the effectiveness of this method and found that the method can reduce transmission delay and improve the system throughput compared with existing algorithms [20,21].

In recent years, motion interaction control has been widely applied in various fields, and the research on motion interaction control has also become a popular research direction. Golparvar et al. used graphene-embedded eye drive receptors as the basis for constructing a human–computer interaction model based on electrooculography and verified the validity of the model and found that the model completed more than 85% of automatic eye movement detection, which was applied to practical application scenarios, and this research helped to advance the development of wearable electronic devices [22]. To explore the integration mechanism between the sensory system and the motor system during finger movement, Wasaka et al. proposed to use somatosensory-evoked potentials and magnetic fields to record the data when the tasker performed the hand movement, and this experiment found primary somatosensory cortex activity distinctly characterized, which may be the basis of the neural mechanism related to finger dexterity [23]. To ensure the rehabilitation robot’s safety, Mancisidor proposed to train the rehabilitation robot for safety using an assistive, corrective, and resistance framework and validated the effectiveness of the trained rehabilitation robot, which was found to operate well in terms of adaptability, robustness, and safety in terms of force control [24]. To improve the human–robot interaction movement stability, Zhuang proposed to construct a musculoskeletal model based on electromyography, and the model validity was verified, and it was found that the auxiliary moment, tracking error, and sharpness value of the model were lower. It improved the stability of movement and was applied to the auxiliary support of patient rehabilitation [25]. To study the functional neural matrix of reward processing and inhibitory control in patients with impulse control disorder, Paz-Alonso et al. proposed a controlled brain–behavior correlation experiment, and the results found that PD patients with impulse control disorder showed excessive activation of the right regional network, including the subthalamic nucleus, which is closely related to the severity of impulse control disorder [26]. In order to improve the quality of life of patients with limb loss, Dantas et al. proposed a decoder based on four kinds of competing muscular intention decoding methods, and upon evaluating the performance of the four decoding methods, found that the performance of the decoder based on a layer perceptron and convolution neural network was optimal, whereby it enabled more accurate and more natural control of a prosthetic hand [27].

In summary, deep reinforcement learning algorithms currently have great application prospects in a variety of fields. To improve the motion fidelity of the motion control model and realize its adaptive function in environmental interactions, this study used a deep reinforcement learning algorithm to improve the model to realize its function of self-adaptation to complex environments.

## 3. Construction of Motor Interaction Control Model Based on Muscle Force Model and Deep Reinforcement Strategy

To improve the motion realism of the motion control model, this study proposed to construct a human motion control model based on the muscle force model and the stage particle swarm algorithm. In addition, based on the human body control model, this research also constructed an environment-oriented intensive interactive motion control model to realize the environmental adaptation and autonomous decision-making function of the motion control model.

### 3.1. Human Motion Control Model Based on Muscle Force and Stage Particle Swarm Optimization

**Figure 1:**
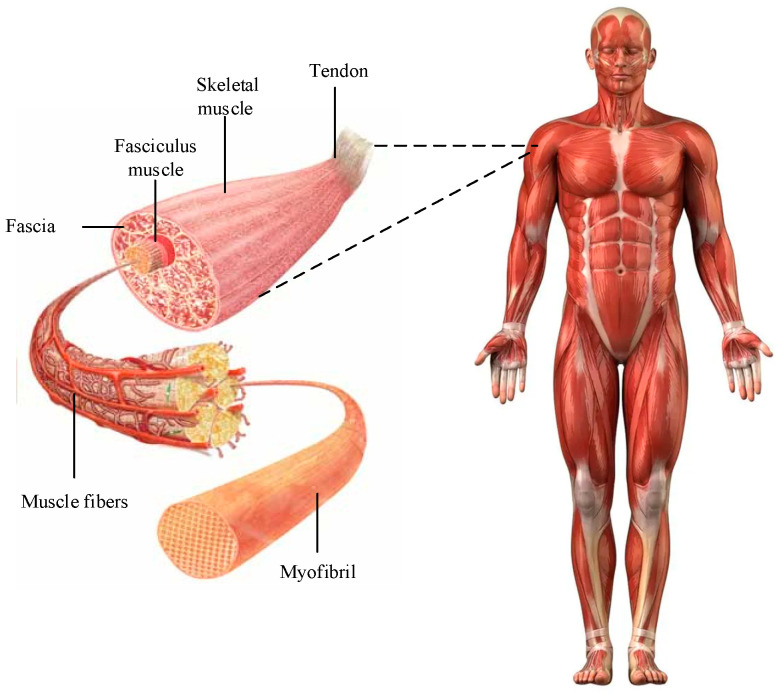
The composition of human skeletal muscle.

To realistically simulate and control the forces and moments of human motion, this study combined biomechanics with optimization algorithms to construct a human motion control strategy based on muscle force and phase particle swarm optimization. The realization of human motion cannot be achieved without bones and muscles. Therefore, this study first constructed a human muscle force model based on the physiological anatomy. The model considers the human body as a skeleton-connected multi-rigid-body system and simulates real human body movements by modeling the limb structure and skeletal muscle mechanics of the real human body. Skeletal muscles in the human body drive bone and joint movements through muscle contraction and relaxation, and the composition of skeletal muscles is shown in Figure 1 [28,29].

Skeletal muscle consists of muscle fibers, as shown in Figure 1. When the nervous system sends a signal, the muscle fibers contract, causing the bones to move. This contraction is produced by the interaction of actin with myosin in myogenic fibers, and the higher the activity value of myogenic fibers, the stronger their contraction force. The human body contains 639 muscles. Due to space limitations, this study took human lower limb movement as the object of movement simulation and constructed a muscle force model of lower limb walking movement. The muscle force in the physiological anatomy of the human body cannot be directly measured, so this study utilized the Hill muscle force three-element model to simulate the muscle contraction force. In the Hill muscle force model, muscles and tendons are regarded as contractile elements, series elastic units, and parallel elastic units [30]. The tandem elastic unit can attach elasticity to the contractile element, while the combination of the contractile element and the tandem elastic unit can act as an actively elastic–contractile element. The expression for calculating the force of a tendon actively performing a contraction is shown in Equation (1):

(1) $$F^{T}=\left(F^{CE}+F^{PE}\right)\cos\vartheta$$

In Equation (1), $F^{T}$ represents the active contraction force of the tendon. $F^{CE}$ represents the contraction force of the contractile element. $F^{PE}$ represents the contraction force of the parallel elastic unit. ϑ represents the angle between the muscle fibers and the direction of the muscle fibers, which is also known as the pinnation angle. The pinnation angle is affected by the length of the muscle fibers, and its calculation is shown in Equation (2):

(2) $$\vartheta \left(t\right)=\arcsin\left(\frac{L^{M_{0}}\sin\vartheta_{0}}{L^{M}}\right)$$

**Figure 2:**
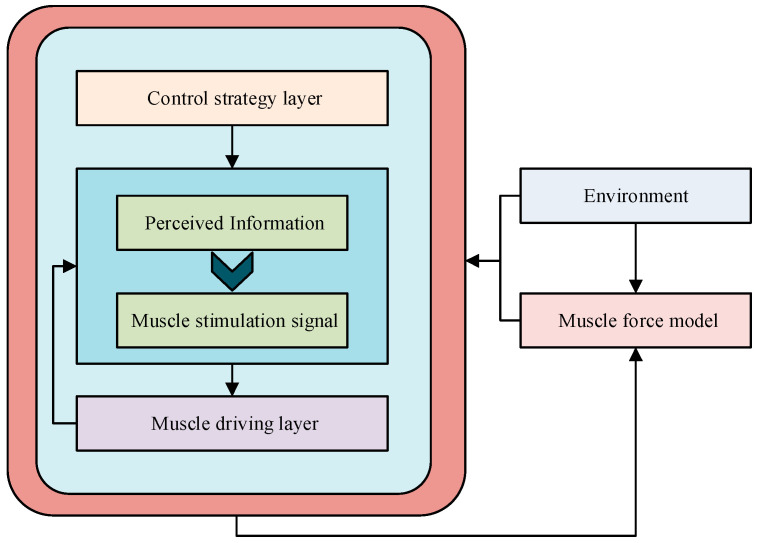
Motor control model based on neural reflexes.

In Equation (2), $\vartheta \left(t\right)$ denotes the pinnation angle after the contraction of the t moment. $L^{M_{0}}$ denotes the optimal isometric contraction length of the muscle fiber. $\vartheta_{0}$ denotes the angle between the fiber and the fiber direction when the fiber is relaxing. $L^{M}$ denotes the length of the muscle fiber when it is t. However, the Hill model is only a simplified version of the muscle force simulation model. To improve the accuracy of muscle force movement simulation, this study also considered the human nerve reflex movement based on Hill’s muscle force model to construct a motor control model. The basic architecture of the model is shown in Figure 2.

**Figure 3:**
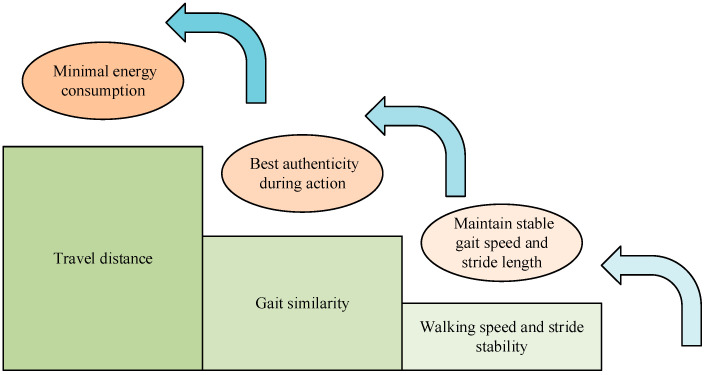
Improved motion control model based on the SPSO algorithm.

In Figure 2, this study divides the motor control model into a strategy control layer, a spinal reflex layer, and a muscle-driven layer. The strategic control layer is located in the highest layer of motor control and is mainly responsible for decision making and planning the overall strategy of the movement. The spinal reflex layer is located in the middle layer of the motor control model, which is mainly responsible for realizing fast muscle contraction and posture adjustment by processing sensory information and its mapping onto muscle excitation signals. The muscle actuation layer is located at the lowest level of motor control and is responsible for performing specific muscle contraction actions. Although this motor control model analyzes motor control strategies at multiple levels, the complexity of the neural reflexes has led to a substantial increase in the parameters involved. Therefore, the study utilized SPSO to optimize the parameters of this motor control model. The SPSO algorithm combines the idea of group intelligence and phased optimization to effectively search the parameter space and find the optimal solution by optimizing the parameters of the strategy control layer, the spinal reflex layer, and the muscle-driven layer. The improved motion control model based on the SPSO algorithm proposed in this study is shown in Figure 3.

In Figure 3, this study divides the objectives of the motion control model into three stages, which are minimizing the energy consumption during the action, optimizing the realism during the action, and keeping the gait speed and step length stable. In the first stage, this study takes the walking distance as the target parameter, and its energy consumption is calculated for the specified walking distance, and the energy consumption calculation is shown in Equation (3):

(3) $$F^{\prime }=d-\alpha E$$

In Equation (3), $F^{\prime }$ denotes the active force produced by the muscles in the walking state. d denotes the walking distance. E denotes the energy consumption. α denotes the energy consumption weight. In the second stage, the study takes the similarity between the simulated gait and the real gait as the target parameter, which is calculated as shown in Equation (4):

(4) $$\left\{\begin{array}{c} C_{ang}=\omega_{hip}^{c}corr_{hip}+\omega_{kne}^{c}corr_{kne}+\omega_{ank}^{c}corr_{ank}\\ C_{poi}=\frac{1}{N}\sum_{n=1}^{N}\left|p_{n}-\hat{p_{n}}\right|\\ F^{\prime }=d-\alpha E+\beta C_{ang}-\gamma C_{poi} \end{array}\right.$$

In Equation (4), $C_{ang}$ denotes the joint trajectory correlation. $C_{poi}$ denotes the joint point gap. $corr_{hip}$ denotes the hip joint correlation. $corr_{kne}$ denotes the knee joint correlation. $corr_{ank}$ denotes the ankle joint correlation. $\omega_{hip}^{c}$, $\omega_{kne}^{c}$, and $\omega_{ank}^{c}$ are the weighting coefficients of 0.35, 0.35, and 0.3, respectively. N denotes the number of key points. $p_{n}$ denotes the simulated key points. $\hat{p_{n}}$ denotes the real key points. In the third stage, this study utilizes the speed and length of the simulated step as the evaluation index of stability, which is calculated as shown in Equation (5):

(5) $$\left\{\begin{array}{c} C_{spd}=-\left|v_{des}-v\right|\\ C_{leth}=-\left|sl_{des}-sl\right|\\ F=d-\alpha E+\beta C_{ang}+\delta C_{spd}+\varepsilon C_{leth} \end{array}\right.$$

In Equation (5), $v_{des}$ denotes the expected step speed, $sl_{des}$ denotes the expected step length, and δ and ε denote the weighting coefficients of the step speed and step length, respectively.

### 3.2. Enhanced Environment-Oriented Interactive Motion Control Modeling

**Figure 4:**
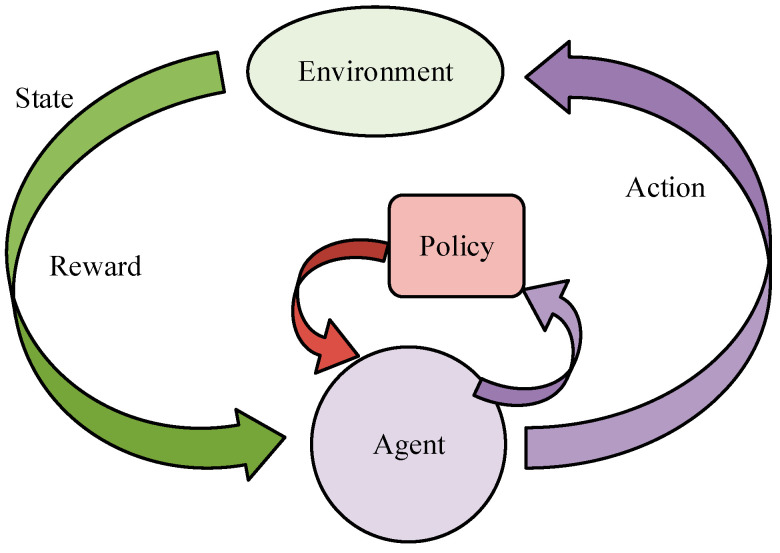
The basic framework of reinforcement learning.

After completing the construction of the motion control model, this study applied it specifically in practice. However, the actual environment is complex and changing. According to the changes in the actual environment, the real-time processing of information and realizing autonomous decision making are also urgent problems to be solved [31,32]. A reinforcement learning algorithm, as a kind of adaptive algorithm, can realize the optimization of an action strategy by learning the reward signal obtained from the environment [33,34]. Therefore, this study proposed to construct an environment-oriented reinforced interactive motion control model using a reinforcement learning algorithm. The basic framework of reinforcement learning is shown in Figure 4.

**Figure 5:**
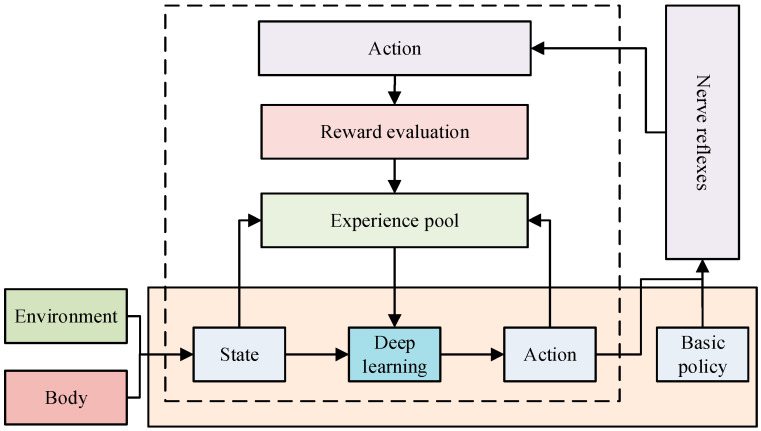
Basic framework of the enhanced motion control model based on environmental interactions.

In Figure 4, reinforcement learning and framework components include five elements: the environment, state, action, reward, and intelligent body. Among them, the intelligent body is the ontology of reinforcement learning and the core of reinforcement learning to realize autonomous decision making. It can learn the optimal strategy by maximizing the accumulated rewards. In motor interaction control, the intelligent body can choose actions according to the current state of the muscle force model, continuously interact with the environment through the action, evaluate the value of learning rewards after executing actions, and continuously try until the intelligent body can gradually master the optimal strategy. Therefore, this study applied reinforcement learning to the motor control model and constructed a reinforcement motor control model based on environmental interactions. Figure 5 shows the model’s basic framework.

In Figure 5, the enhanced motion control model based on environmental interactions includes a base strategy module for invariant environments, a stochastic strategy module for changing environments, and an experience pool module for learning and training. For the base strategy module, the optimized stage particle swarm update calculation is shown in Equation (6):

(6) $$\left\{\begin{array}{c} x_{i}(n+1)=x_{i}(n)+v_{i}(n+1)\\ v_{i}(n+1)=\omega *v_{i}(n)+c_{1}*(x_{g}-x(n))*Rand[0:1]+c_{2}*(x_{p}-x(t))*Rand[0:1] \end{array}\right.$$

In Equation (6), $x_{p}$ is the individual optimal position. $x_{g}$ is the global optimal position. ω represents the inertia weight. $c_{1}$ represents the social factor. $c_{2}$ represents the cognitive factor. At this time, the objective function of particle swarm updating in the process of basic strategy optimization is shown in Equation (7):

(7) $$R_{0}=e^{E_{v}+E_{p}+E_{e}},no\;falls\;down$$

In Equation (7), $E_{v}$ denotes the return value of the simulated human movement speed. $E_{p}$ denotes the return value of the simulated human position. $E_{e}$ denotes the return value of the simulated human movement energy consumption. When the environment changes, the model adjusts the behavioral strategy of the human body through the deep learning algorithm actor–critic. The main update expression for the stochastic strategy is shown in Equation (8):

(8) $$\nabla_{\theta }J(\theta )=E_{s\sim p^{\pi },a\sim \pi_{\theta }}\left[\nabla_{\theta }\log\pi_{\theta }(a\left|s\right.)Q^{\pi }(s,a)\right]$$

In Equation (8), a denotes the behavior, s denotes the state, and Q denotes the strategy evaluation. The average gradient estimation of the strategies using the empirical pool is shown in Equation (9):

(9) $$\left\{\begin{array}{c} \nabla_{\theta }U(\theta )\approx \frac{1}{m}\sum_{i=1}^{m}\nabla_{\theta }\log P(\tau ;\theta )R(\tau )\\ R(\tau )=\sum_{t=0}^{H}R(s_{t},u_{t}) \end{array}\right.$$

**Figure 6:**
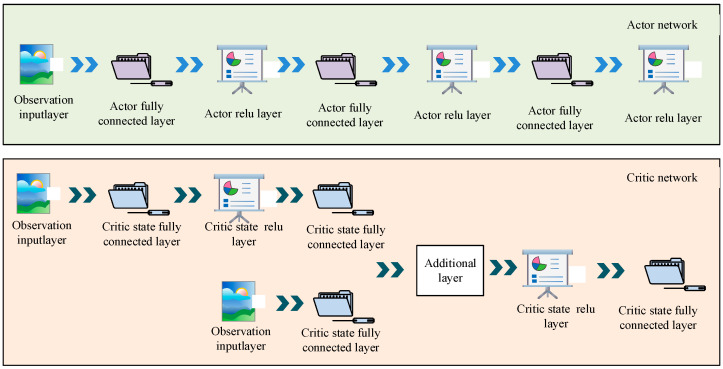
Basic architecture of the experience pooling module based on DDPG.

In Equation (9), τ denotes the behavioral sequence. θ denotes the direction parameter. τ denotes the trajectory. P(τ;θ) denotes the probability of the trajectory’s occurrence. R(τ) denotes the trajectory’s return. The gradient strategy can make the trajectory return the highest evaluator, increase the probability of a high return estimation, and then improve the training speed. Therefore, in the experience pool module, this study used the deep deterministic policy gradient (DDPG) algorithm to optimize it. The basic architecture of the DDPG-based experience pooling module is shown in Figure 6.

The DDPG algorithm also includes two modules, the actor network and the critic network, and its training process includes two phases: policy update and value function update. In the strategy update phase, DDPG calculates the gradient of the strategy based on the current state and the action output from the strategy function and updates the strategy parameters based on the gradient. In the value function update phase, the DDPG updates the parameters of the value function based on the current state, the actions and the rewards fed back from the environment, and based on the loss. The expression for calculating the target value of its information update is shown in Equation (10):

(10) $$\eta =r_{t}+\gamma Q^{w}(s_{t+1},\mu_{\theta }(s_{t+1}))$$

In Equation (10), η indicates the information update target. μ denotes the policy network. w and θ denote the parameter values to be updated. The updating expression of the parameters is shown in Equation (11):

(11) $$\left\{\begin{array}{c} w_{t+1}=w_{t}+a_{w}\delta_{t}\nabla_{w}Q^{w}(s_{t},\alpha_{t})\\ \theta_{t+1}=\theta_{t}+a_{\theta }\nabla_{\theta }\mu_{\theta }(st)\nabla \alpha Qw(s_{t},\alpha_{t}){|}_{\alpha =\mu_{\theta }(s)}\\ w^{\prime }=\tau \theta +(1-\tau )\theta^{\prime }\\ \theta^{\prime }=\tau w+(1-\tau )w^{\prime } \end{array}\right.$$

In Equation (11), $w^{\prime }$ and $\theta^{\prime }$ denote the updated parameters. The inputs of the motion control model need to consider not only the state characteristics but also the strategy behavior. If the simulated human body falls, its return strategy and function value is 0, and the return function calculation for walking is shown in Equation (12).

(12) $$r(s,a,s^{\prime })=e^{E_{v}+E_{j}}$$

In Equation (12), r denotes the payoff function. $E_{j}$ denotes the moment sum in the joint hyperextension state. The training expression of the strategy evaluation network is shown in Equation (13):

(13) $$\left\{\begin{array}{c} L_{Q}(\theta^{Q})=E_{s_{t},a_{t},r_{t},s_{t+1}\sim D}\left[(Q(s_{t},a_{t})-y_{t})2\right]\\ y_{t}=r_{t}+\gamma Q^{\prime }(s_{t+1},a)\left|a=\mu^{\prime }(s_{t+1})\right. \end{array}\right.$$

In Equation (13), D denotes the experience pool. The policy network training expression is shown in Equation (14):

(14) $$\nabla_{\theta^{\mu }}J={Ε}_{s_{t}\sim D}\left[\nabla_{a}Q(s_{t},a\left|\theta^{Q}\right.){|}_{a=\mu (s_{t})}\nabla_{\theta^{\mu }}\mu (s_{t}\left|\theta^{\mu }\right.)\right]$$

In the DDPG-optimized experience pool, this study selected components of size 106 as the buffer tuples. During the accumulation of the experience pool, the tuple is continuously deposited into the buffer, and when the buffer is full, the old samples in the experience pool are discarded as a way of realizing the parameter update. This method improves the efficiency of sample utilization.

## 4. Empirical Experiments on Motor Interaction Control Model Based on Muscle Force Model and Depth Reinforcement Strategy

To validate the effectiveness of this research’s proposed motor interaction control model based on the muscle force model and deep reinforcement strategy, this study first conducted performance validation on the motion fidelity of the human motion control model. In addition, to verify the environmental adaptive function of the reinforced interaction motor control model, this study conducted performance verification experiments on the model by setting up different obstacle environments for its movement and learning efficiency.

### 4.1. Validation of the Effectiveness of the Human Motion Control Model

**Figure 7:**
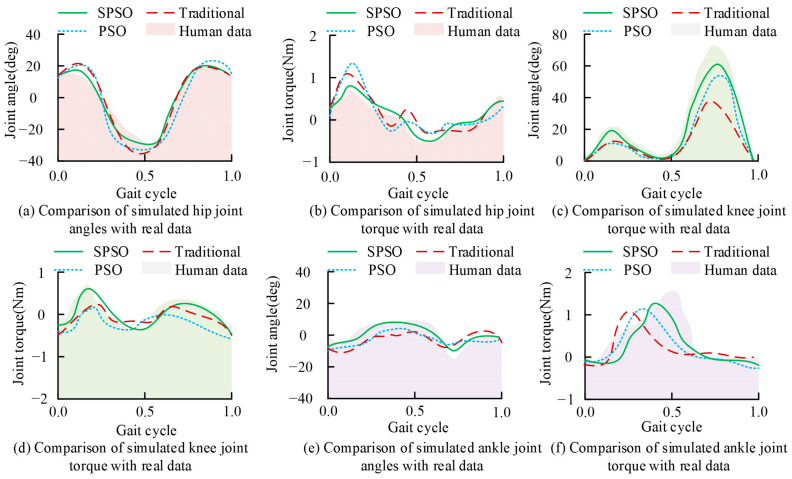
Comparison results of joint angles and joint torque between each model’s simulation data and human body data.

To validate the effectiveness of the proposed motion control model optimized based on the muscle force model and SPSO algorithm, this study compared the performance of the proposed motion control model based on PSO and the traditional motion control model. This study validated the effectiveness of the SPSO motion control model with the gap between the simulated motion data and the real human body data. The evaluation indexes were a joint angle, joint moment, muscle activity, joint trajectory correlation, and muscle activity correlation. Due to the large number of tendon units in the human lower limbs, due to space limitations, only the gluteus (GLU), the femur (VAS), the hamstring (HAM), and the soleus (SOL) were selected as the representatives in this study to determine their muscle activities. The simulation platform used for this study was MATLAB Simulink, the solver was ode 15 s, and the stopping criterion $d_{\lim}$ was set to 25 m. The joint angles and joint moments in the motion data of each comparative model and the data on the real human body are shown in Figure 7.

**Figure 8:**
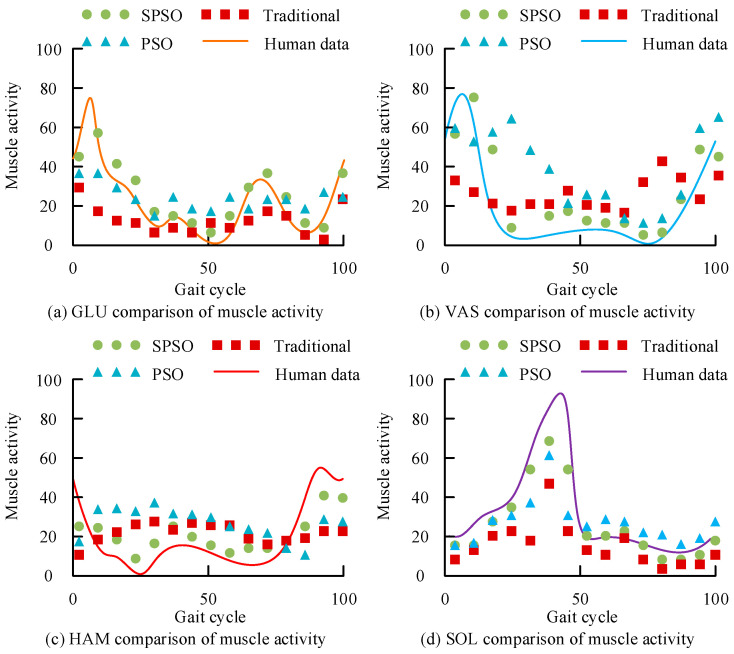
Comparison results of the generated data of each motion control model with the real human EMG data.

Figure 7a,b shows the comparison results of the hip joint angle and moment of each model. The proposed SPSO motion control model had a better fit with the real hip joint motion data, and the maximum error of its angle and moment was 8.6 deg and 0.1 Nm, which better reflected the human motion data. Figure 7c,d shows the comparison results of the knee joint angles and moments for each comparison model. The proposed motion control model had a better fit with the real knee joint motion data, and the maximum errors of its angles and moments were 19.4 deg and 0.4 Nm. Figure 7e,f shows the comparison results of the ankle joint angles and moments for each comparison model, and the proposed motion control model could better represent the human motion data. The proposed motion control model had a better fit with the real ankle joint motion data, and the maximum errors of its angle and moment were 8.7 deg and 0.5 Nm. In summary, the proposed motion control model better simulated the real human body’s joint motion trajectory. The results of the comparison between the data generated by the muscle force model of each motion control model and the EMG data of the real human body are shown in Figure 8.

**Figure 9:**
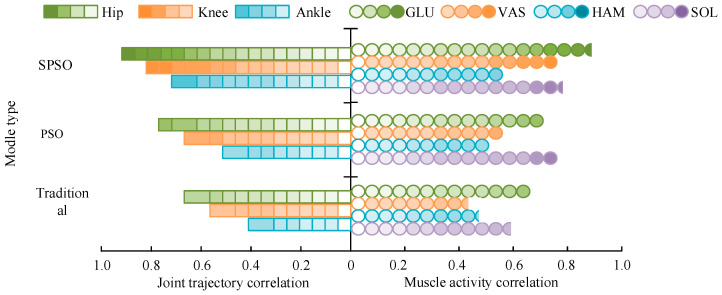
Joint trajectory and muscle activity correlation of each motor control model.

Figure 8a–d shows the GLU, VAS, HAM, and SOL muscle activity data generated by each motion control model, respectively. In Figure 8, the muscle activity generated by SPSO was closer to the real human motion data muscle activity than the other models. The maximum error of GLU muscle activity was 12.6, the maximum error of VAS muscle activity was 9.1, the maximum error of HAM muscle activity was 8.4, and the maximum error of SOL muscle activity was 26.6. Considering the amount of computation, this study carried out a certain amount of simplification on the muscle model, which made the simulation results not 100% fit with the real results. However, summarizing the results, the motion control optimization model proposed in this study had a better simulation performance than the other methods while ensuring the computational amount. The comparison results of the correlation between the joint trajectories and muscle activity of each motion control model are shown in Figure 9.

In Figure 9, the correlation between the joint trajectories and muscle activities generated by the motion control model based on SPSO optimization and the real human body data is better. The correlation of the joint trajectories of the SPSO motion control model was up to 0.90, and the correlation of its muscle activities was up to 0.84. Summarizing the above results, the motion control model using the optimization of SPSO proposed in this study simulated the real human body motion better and with better motion fidelity.

### 4.2. Enhanced Environment-Oriented Motion Interaction Control Modeling

After completing the validation of the motion fidelity of the motion control model, this study also validated the effectiveness of the motion interaction control model using the deep reinforcement strategy. To investigate the autonomous regulation and control performance of the motion interaction control model in the face of complex and variable environments, this study utilized a motion interaction control model based on the classical deep reinforcement learning method (Deep Q-Network (DQN)), the proximal policy optimization (PPO) algorithm, and the traditional motion interaction control model to conduct performance comparison experiments on the motion interaction control model. In this study, steps, slopes, and uneven ground were set as the environmental obstacles, the inertia weight was set to 0.7, and the cognitive factor and social factor were set to 2.1. The comparison indexes were the average distance traveled before 100 falls and the return value of the learning process of each model in different obstacle environments. The simulation platform was MATLAB Simulink. The average walking distance of each model in different obstacle environments is shown in Table 1.

**Figure 10:**
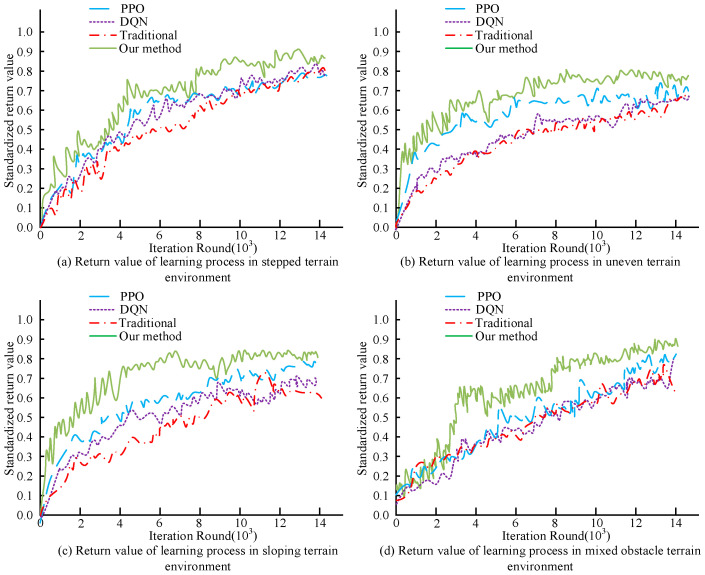
Change curve of the reward value of each model.

In Table 1, the proposed motor interaction control model based on the deep reinforcement strategy better interacted with the complex environments, and its average walking distances were 409 m in a step environment, 472 m on a slope, 434 m in uneven terrain, and 423 m in mixed terrain, which are farther than those of the other models. In summary, the motion interaction control model based on the deep reinforcement learning strategy had better robustness to complex environments and was better applied to real-world scenarios. This study also validated the performance of each model explored in the different obstacle environments, and the change curves of the reward values of each model in learning different scenes are shown in Figure 10.

**Figure 11:**
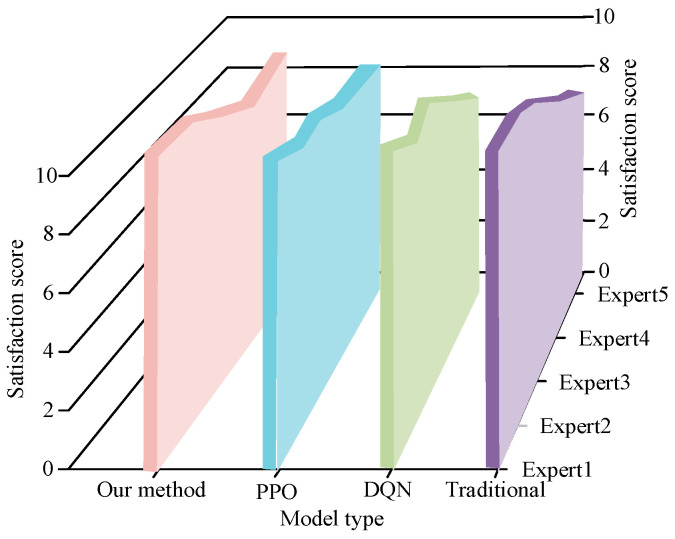
Results of expert satisfaction scores.

Figure 10a–d shows the change curves of the learning reward values in the step, uneven, slope, and hybrid modes, respectively. In Figure 10, the motion control model based on the deep learning strategy proposed in this study exhibits a higher learning efficiency. The desired results were obtained by training 9.0 × 10^3^ times in the step obstacle environment, 8.2 × 10^3^ times in the uneven obstacle environment, 6.9 × 10^3^ times in the slope environment, and 1.1 × 10^3^ times in the hybrid environment, which are lower values than those of the other compared models. Summarizing the results, the training times of the motion control model based on the deep learning strategy were less and its learning efficiency was better. In addition, to further verify the effectiveness of the motion interaction control model using the deep learning strategy proposed in this study, this study evaluated the smoothness, accuracy, and robustness of the output motion of the model in all aspects of satisfaction through expert scoring. The results of the expert satisfaction scores for each model are shown in Figure 11.

In Figure 11, the average score of this research’s proposed motor interaction control model is 8.4, the average satisfaction score of the PPO-based motor interaction control model is 7.9, the average satisfaction score of the DQN-based motor interaction control model is 7.5, and the average satisfaction score of the traditional motor interaction control model is 7.2. To summarize the results, the proposed motor interaction control model using the deep reinforcement strategy had the highest expert score, which indicated that the model had higher smoothness, accuracy, and robustness than the other models and had practical application value. In addition, to further show the movement interaction control model and existing motion interaction control model performance contrast effect, this study also used performance comparison experiments to establish the motion interaction models and compare them with the motion interaction models in references [16,20,23], with the comparison indexes of the mixed terrain walking distance, model control accuracy, motion deviation, and convergence speed. The performance comparison results of each motion control model are shown in Table 2.

In Table 2, the motion interaction model constructed in this study has a walking distance of 423 m, a control accuracy of 92.3%, and a movement deviation of 0.1 N, which are higher than those of the other contrasted models. However, its convergence rate has little advantage compared with the other contrasted models, being only slightly higher than that of the motion interaction model shown in reference [32]. From the above results, compared with the existing motion interaction model, the motion interaction model needed a large number of samples and time to learn to adapt to the environment, and the optimization effect of the training time was insufficient. The reason for this may be that the DDPG optimization strategy was more sensitive to the initial strategy, and when the initial strategy was not selected correctly, its training speed was greatly affected.

## 5. Conclusions

To further improve the motion fidelity of the motion interaction model and realize the self-adaptive function of the motion interaction model to complex environments, this study proposed to construct a human motion control model based on the muscle force model and the SPOS algorithm. Based on this, an environment-oriented strengthened motion interaction control model was constructed by using the deep reinforcement learning algorithm. The simulation of the proposed muscle force model-based human movement control strategy model found that the joint trajectory correlation and muscle activity correlation of this model were higher than those of the other comparative models, and its joint trajectory correlation was up to 0.90, and its muscle activity correlation was up to 0.84. In addition, this study also verified the effectiveness of the environment-oriented reinforcement movement interaction control model and found that the model achieved the highest joint trajectory correlation and muscle activity correlation at a walking distance of 4.5 m in uneven terrain. In addition, the effectiveness of the environment-oriented reinforced motor interaction control model was also verified, and it was found that the walking distance of this model in uneven terrain was 434 m, and in mixed terrain, it was 423 m, which was farther than those of the other models. Moreover, the model obtained the desired results by training 8.2 × 10^3^ times in uneven terrain, while it needed to be trained 1.1 × 10^3^ times in a mixed-obstacle environment, which was more efficient than other models. In summary, the proposed motor interaction control model using the muscle force model and deep reinforcement strategy had higher motion fidelity than the traditional model, and it realized autonomous decision making and adaptive control in the face of complex environments. The constructed motion control model can be applied in practice, such as in medical rehabilitation, robot motion control, and virtual character motion control. The motion control model constructed in this research can provide help for the treatment of patients with motor dysfunction and be transplanted into a robot control system to improve the motion nature of anthropomorphic robots and improve the real-time and realistic performance of virtual characters. However, there are some limitations to this study. Considering the amount of computation and the computational cost, the muscle force model constructed in this study has a simplification step, which limits the simulation motion performance. The future research direction is to further improve the muscle force model and enhance the simulation realism based on controlling the computational cost.

## Figures and Tables

**Table 1:** Average walking distance of each model in different obstacle environments.

Optimized Control Method	Distance Traveled			
	Footstep	Slope	Uneven	Admixture
Our method	409	472	434	423
DQN	283	247	207	234
PPO	221	204	194	152
Traditional	156	174	143	121

**Table 2:** Performance comparison results of each motion control model.

Optimized Control Method	Admixture	Control Accuracy	Action Deviation	Convergence Rate
Our method	423 m	92.3%	0.1 Nm	9.0 × 10^3^
[14]	314 m	91.4%	0.2 Nm	9.4 × 10^3^
[16]	284 m	87.7%	0.3 Nm	9.1 × 10^3^
[17]	311 m	90.5%	0.2 Nm	9.5 × 10^3^

## Data Availability

Dataset available on request from the authors.
